# Population genomics of Aosta Valley rye landraces reveals genetic structure and diversity patterns relevant for germplasm conservation

**DOI:** 10.3389/fpls.2026.1901479

**Published:** 2026-09-03

**Authors:** Ezio Portis, Edoardo Vergnano, Mauro Bassignana, Matteo Martina

**Affiliations:** 1 DISAFA Plant Genetics, University of Turin, Grugliasco, Italy; 2 Institut Agricole Régional, Aosta, Italy

**Keywords:** genetic diversity, germplasm conservation, K-seq, landraces, population genomics, population structure, *Secale cereale*, SNP markers

## Abstract

**Introduction:**

Rye (*Secale cereale* L.) is a cereal crop well adapted to marginal environments, where traditional populations represent valuable reservoirs of genetic diversity. In the Aosta Valley (NW Italy), several local rye populations have historically been maintained under small-scale farming systems, but their genetic structure has not yet been investigated using genome-wide approaches.

**Methods:**

We performed a high-resolution population genomic analysis of twelve rye populations using a K-seq genotyping approach. A genome-wide SNP dataset was generated and used to assess genetic diversity and population structure through complementary multivariate, distance-based, and model-based analyses.

**Results:**

Substantial genetic variation was detected within populations (H_T_ = 0.354), together with a moderate level of genetic differentiation among populations (G_ST_ = 0.254). Populations collected in the 1980s showed consistently higher genetic diversity and differentiation (H_T_ = 0.392; G_ST_ = 0.311) than populations collected in 2000 (H_T_ = 0.306; G_ST_ = 0.180), suggesting a reduction in genetic variability in more recent germplasm. Multivariate and clustering analyses identified distinct genetic groups and clusters of closely related populations, revealing a complex genetic structure shaped by both differentiation and admixture.

**Discussion:**

These findings provide an updated view of the genetic architecture of Aosta Valley rye populations and underline the importance of conserving genetically diverse and differentiated populations. More broadly, the study demonstrates the utility of K-seq for generating informative SNP datasets and investigating population structure in rye, supporting its application to the characterization and management of plant genetic resources.

## 1 Introduction

Rye (*Secale cereale* L.) is a cereal crop well adapted to marginal environments, including mountainous and low-input agroecosystems, where it has historically played a key role in sustaining local agriculture (Sardella et al., 2023; Raggi et al., 2024; Miedaner, 2025). Its tolerance to low temperatures, poor soils and abiotic stresses has made rye a staple crop in alpine and sub-alpine regions of Europe, where traditional populations have been maintained through farmer-managed seed systems (Peratoner et al., 2016; Båga et al., 2022; Gentili et al., 2024).

These populations represent an important component of agro-biodiversity, as they harbor genetic variation relevant for adaptation to changing environmental conditions and for future breeding programs. However, the progressive abandonment of mountain agriculture and the replacement of traditional varieties with modern cultivars have contributed to genetic erosion and loss of locally adapted germplasm (Marone et al., 2021; Raggi et al., 2022; Lazaridi et al., 2024). Recent studies based on next-generation sequencing (NGS) approaches have highlighted the narrowing of the genetic base in cultivated rye and the importance of preserving local populations as reservoirs of genetic diversity (Adamo et al., 2021).

In the Western Alps, several surveys have been conducted to recover and characterize remnant rye populations still maintained by local farmers. A recent genomic study based on ddRAD sequencing investigated rye diversity across a broad alpine area, revealing patterns of genetic structure and suggesting a progressive homogenization of the local gene pool (Adamo et al., 2021). While this work provided an important regional overview, fine-scale analyses focusing on well-defined local germplasm collections remain limited. In the Aosta Valley (NW Italy), rye was historically the main cereal crop, and several populations have been preserved in small-scale farming systems. A previous study based on AFLP markers provided a first assessment of the genetic diversity and structure of these populations, highlighting both significant within-population variability and differentiation among populations (Portis et al., 2011). However, a comprehensive genome-wide characterization of this germplasm is still lacking.

The advent of NGS technologies has enabled the transition from fragment-based markers to high-density SNP datasets, greatly enhancing the power of population genetic analyses. Among reduced-representation approaches, K-seq uses Klenow-based primer extension and oligonucleotides selected from abundant genomic k-mers to generate a reproducible, species-tailored representation of the genome. Unlike GBS and ddRAD-seq, this approach does not depend on restriction-enzyme digestion and allows the genomic representation to be adjusted through primer selection (Martina et al., 2023).

In this study, we applied a K-seq approach to perform a high-resolution genomic characterization of twelve rye populations from the Aosta Valley, including both historical (1980s) and more recent (2000) collections. The objectives were to (i) assess the level of genetic diversity within and among populations, (ii) investigate population structure and genetic relationships, and (iii) provide insights relevant for the conservation and management of local germplasm. By focusing on a well-defined historical collection, this study complements regional surveys and contributes to a deeper understanding of rye genetic resources in alpine environments.

## 2 Materials and methods

### 2.1 Plant material and DNA sampling

A total of twelve rye (*Secale cereale* L.) populations originating from the Aosta Valley (located in North West of Italy) were analyzed in this study. The plant material comprised two historical collections: five populations collected in the 1980s by researchers from Agroscope Changins–Wädenswil (ACW, Switzerland) and seven populations collected approximately two decades later by the Institut Agricole Régional (IAR, Aosta, Italy) from small-scale local farms where seed was traditionally recycled on-farm. All populations originated from the Aosta Valley, although the two collections did not necessarily include the same sampling localities. Therefore, comparisons between the ACW and IAR groups should be interpreted as comparisons between two historical collections from the same regional area, rather than as repeated sampling of identical sites. Population names, sample identifiers, collection groups, collection years and locality information are detailed in **Supplementary Table 1**.

Each population was represented by multiple individuals (16 plants per population), sampled to capture the within-population variability typical of this allogamous species. Genomic DNA was extracted from young leaf tissue following adapted CTAB extraction (Li et al., 2024), and subsequently stored at −80 °C. For the present study, archived DNA samples were retrieved and used for genotyping. A total of 192 individuals were included in the analysis, ensuring a balanced representation of all populations and providing a robust dataset for genome-wide SNP-based population genetic analyses. Throughout the manuscript, populations are grouped according to their collection source and period as ACW (collected in the 1980s) and IAR (collected in 2000).

### 2.2 K-seq library preparation and sequencing

Primer sets were identified on the rye (*Secale cereale* L.) reference genome using the Primer Explorer 2 pipeline, following the approach described for K-seq by Mata-Nicolás et al. (2021) and Ziarsolo et al. (2021). Candidate oligonucleotide sets were generated from the most abundant k-mers showing 35–75% GC content and were subjected to in silico PCR to estimate the number of predicted amplification products. Primer combinations were then selected according to their expected genome representation and suitability for genotyping, as previously described by (Martina et al., 2023). Based on these criteria, the primer combination CAAGCAAA–ATGATGTG was selected and used for K-seq library preparation.

Briefly, genomic DNA was denatured and annealed with forward primers at 37 °C, and first-strand synthesis was carried out using the large Klenow fragment of DNA polymerase I (New England Biolabs, MA, USA). After enzyme inactivation at 75 °C, residual single-stranded DNA was digested with Exonuclease I (New England Biolabs, MA, USA) at 37 °C and subsequently inactivated at 80 °C. The same procedure was then repeated with reverse primers. The resulting products were used as template for a 15-cycle enrichment PCR with dual-indexed Illumina Nextera primers. Indexed samples were pooled, and libraries were size-selected in the 300–700 bp range by gel excision and purified using the E.Z.N.A. Gel Extraction Kit, following the manufacturer’s instructions.

### 2.3 SNP calling and data filtering

Libraries were sequenced on an Illumina NovaSeq 6000 platform (Illumina, San Diego, CA, USA) using 150-bp paired-end reads. After demultiplexing, raw reads were trimmed and quality-filtered with *fastp* (Chen et al., 2018). Cleaned reads were aligned to the *Secale cereale* Lo7 reference assembly lpSecCere.Lo7.IPK.v3 (NCBI assembly accession GCA_965641915.1) (Rabanus-Wallace et al., 2021) using BWA-MEM (Li and Durbin, 2009). SNP calling was performed with *bcftools mpileup* and *bcftools call* (Danecek et al., 2021). Variants were filtered by retaining sites with a mean read depth across genotyped samples of at least 20 [MEAN(FMT/DP) ≥ 20], a mean genotype quality across genotyped samples greater than 20 [MEAN(FMT/GQ) > 20], no more than 20% missing genotypes (F_MISSING ≤ 0.20), and a minor allele frequency of at least 0.05 (MAF ≥ 0.05), following the filtering procedure previously applied by Martina et al. (2023). Only SNPs overlapping annotated genic regions were retained for downstream analyses using *bedtools intersect* (Quinlan and Hall, 2010).

### 2.4 Genetic diversity and population structure analysis

Population structure and genetic differentiation were investigated using a SNP dataset derived from K-seq genotyping, represented as VCF-based variant data (**Supplementary Table 1**). Analyses were performed across the Aosta Valley rye populations using complementary diversity-based, distance-based, model-based, and multivariate approaches.

Genetic diversity parameters were calculated from allele frequencies across the retained biallelic SNP dataset. For each locus, gene diversity was calculated as $H=1-\sum p_{i}^{2}$, where *p_i_* represents the frequency of allele *i*. Total gene diversity (H_T_) was calculated from pooled allele frequencies for each population or population group, whereas within-population gene diversity (H_S_) was calculated as the arithmetic mean of gene diversity values estimated across the component populations. Genetic differentiation was estimated as G_ST_=(H_T_−H_S_)/H_T_. The observed number of alleles (n_a_), effective number of alleles (n_e_), and Shannon’s information index (I) were calculated from allele frequencies across the same retained SNP dataset. The percentage of polymorphic loci (PL) was calculated as the proportion of retained loci that were polymorphic within the corresponding population or population group. Loci that were monomorphic within a given population or population group were retained in all diversity calculations and contributed zero diversity for that population or group.

Pairwise G_ST_ and Nei’s genetic identity values were calculated among populations from allele frequencies across the retained SNP dataset (Nei, 1973), and the resulting population-by-population matrix was visualized as a heatmap to summarize genetic relationships and differentiation across the dataset. Nei’s genetic distance was calculated as D=-ln (I_N_), where I_N_ is Nei’s genetic identity, and was used to construct the Neighbour-Joining (NJ) dendrogram. Population relationships were additionally explored by UPGMA clustering based on pairwise G_ST_ values (Sokal and Michener, 1958; Saitou and Nei, 1987). These analyses were interpreted as distance-based summaries of population relationships.

Model-based inference of ancestry coefficients was performed using sparse non-negative matrix factorization implemented in the R package LEA (Frichot et al., 2014), using R (R Core Team, 2026). Genotype data were converted to the appropriate input format, and 15 replicate runs were performed for each K value from 1 to 12. For each K, the replicate with the lowest cross-entropy was retained, and cross-entropy values were compared across K values. The overall minimum cross-entropy was observed at K = 11. Because higher K values resulted in progressively finer subdivision of ancestry components, K = 7 was additionally retained as a lower-complexity solution for visualization and biological interpretation of the major ancestry patterns.

To complement model-based clustering with a distance-based multivariate framework, Principal Coordinates Analysis (PCoA) was performed in R on scaled Euclidean distances calculated from the genotype dosage matrix. The dimensionality of the genetic variation was assessed using a Cattell scree plot based on eigenvalues (**Supplementary Figure 1**), and the proportion of variation explained by each coordinate was reported on the figure axes. All statistical analyses and figure generation were conducted in R, using ggplot2 and associated visualization packages. Final figures were exported as publication-quality vector graphics.

## 3 Results

### 3.1 SNP discovery and dataset overview

**Figure 1:**
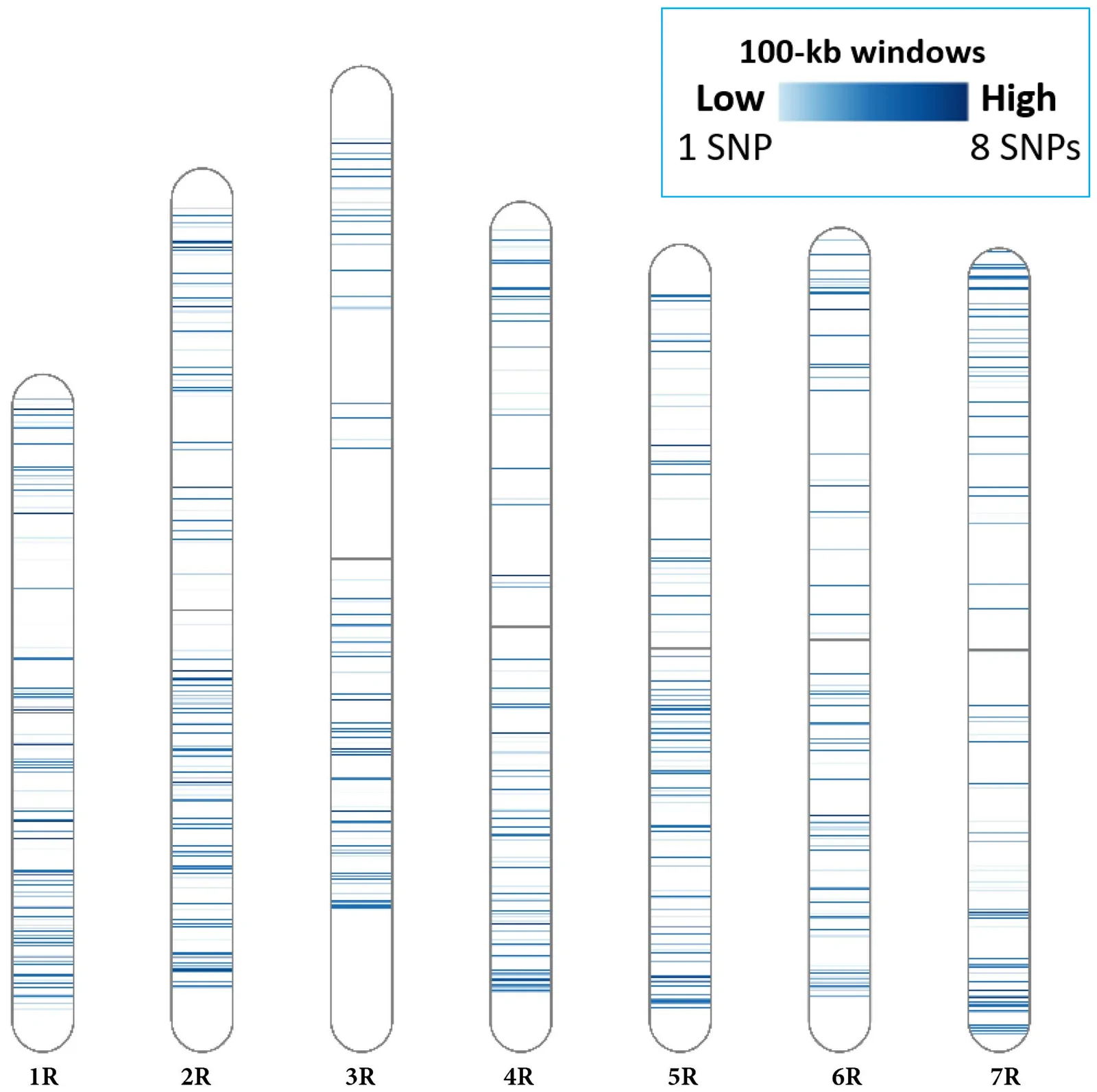
Genome-wide distribution of SNP markers obtained by K-seq genotyping in rye. Chromosomal ideograms display SNP abundance along chromosomes 1R–7R, calculated in 100-kb windows. Color intensity reflects SNP density within each window, ranging from 1 SNP (low) to 8 SNPs (high). The figure shows a broad distribution of markers across all chromosomes, with heterogeneous local marker density.

High-throughput sequencing generated 234,993,556 reads across 192 samples, corresponding to 117,496,778 read pairs. Samples yielded an average of 611,962 ± 87,480 read pairs, ranging from 405,462 to 944,396 read pairs per sample. Sample-level sequencing statistics are provided in **Supplementary Table 1**. Initial variant calling identified ~45,000 raw SNPs genome-wide, of which 18,741 were located in genic regions. After filtering, 2,332 high-quality genic SNPs were retained for population structure analyses (**Supplementary Table 1**). The filtered markers were distributed across all seven rye chromosomes, although marker density varied among chromosomes and genomic regions (**Figure 1**). Chromosome-level SNP counts, densities and terminal-region counts are reported in **Supplementary Table 1**. Approximately 41% of the initially identified variants were located within annotated genic regions.

### 3.2 Genetic diversity and multivariate analysis

Genetic diversity estimates based on the filtered SNP dataset revealed substantial variation within populations (**Table 1**). Across all populations, total gene diversity (H_T_) was 0.354. When considering groups separately, populations collected in the 1980s (ACW) showed higher diversity (H_T_ = 0.392) compared to those collected in 2000 (IAR) (H_T_ = 0.306). A similar trend was observed for all other diversity parameters, including the percentage of polymorphic loci (PL), the observed (na) and effective number of alleles (ne), and Shannon’s information index (I), which were consistently higher in ACW populations than in IAR populations.

**Table 1:** Summary of genetic diversity parameters for each rye population and population groups.

Population	PL	n _a_	n _e_	H _T_	I
**Rhêmes St. Georges**	78.7	1.78	1.51	0.309	0.456
**Sarriod**	79.8	1.80	1.50	0.289	0.449
**Valle d ’ Aosta-46**	77.7	1.79	1.52	0.294	0.448
**Vens-1**	79.2	1.86	1.57	0.291	0.452
**Vens-2**	84.0	1.79	1.53	0.299	0.447
**AC W 1980 populations**	**100.0**	**2.00**	**1.71**	**0.392**	**0.564**
**Arnad**	72.4	1.71	1.39	0.256	0.407
**Brusson**	73.5	1.69	1.44	0.246	0.400
**Champorcher**	67.1	1.70	1.55	0.235	0.352
**Fénis**	74.2	1.68	1.41	0.241	0.373
**Morgex**	69.9	1.74	1.42	0.225	0.401
**Pontboset**	66.1	1.66	1.36	0.219	0.318
**Sarre**	72.1	1.69	1.47	0.252	0.382
**IA R 2000 populations**	**95.7**	**1.96**	**1.55**	**0.306**	**0.490**
**All populations**	**100**	**2.00**	**1.61**	**0.354**	**0.531**

PL, percentage of polymorphic loci; na, observed number of alleles; ne, effective number of alleles; H_T_, total gene diversity; I, Shannon’s information index. Population groups correspond to ACW (1980s collections), IAR (2000 collections), and the complete dataset.

At the single-population level, H_T_ values ranged from 0.219 (Pontboset) to 0.309 (Rhêmes St. Georges), indicating moderate variation among populations. The percentage of polymorphic loci (PL) varied from 66.1% (Pontboset) to 84.0% (Vens-2), further supporting the heterogeneous nature of these traditional rye populations.

**Figure 2:**
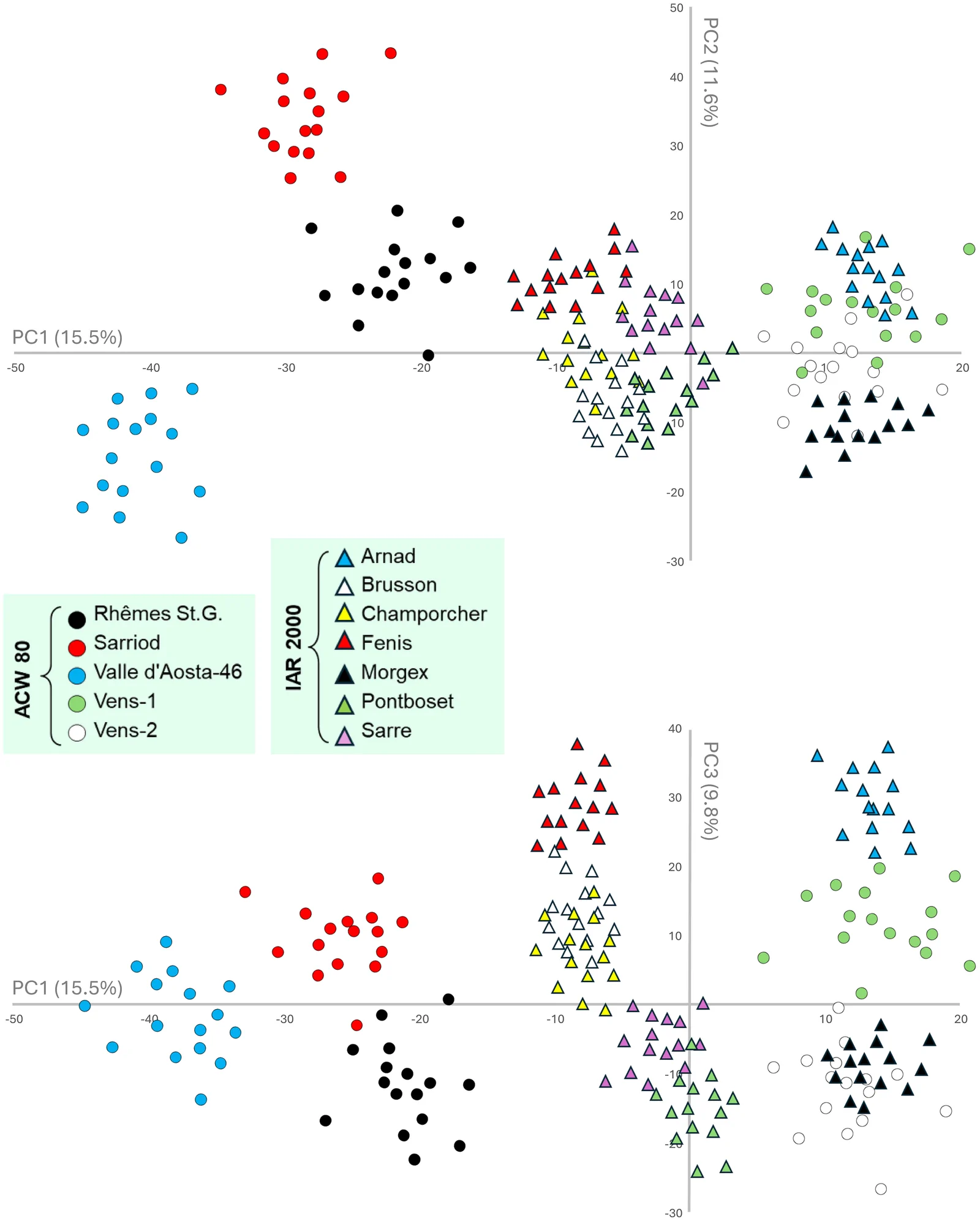
Principal Coordinates Analysis (PCoA) of genetic relationships among rye individuals. Scatterplots of the first vs second (top) and first vs third (bottom) coordinates show the genetic structure of the analyzed populations. Individuals are grouped by population; populations collected in the 1980s (ACW) are indicated by circles, whereas populations collected in 2000 (IAR) are represented by triangles.

Principal Coordinates Analysis (PCoA) provided a clear overview of the genetic relationships among individuals and populations (**Figure 2**). The first two coordinates allowed the identification of well-defined patterns of genetic structure, with individuals generally clustering according to their population of origin.

In particular, the first and second coordinates (explaining respectively 15.5% and 11.6% of the total variation) clearly separated individuals belonging to the ACW populations Rhêmes St. Georges, Sarriod and Valle d’Aosta-46 from the remaining populations. Along the first coordinate, two broad population groupings were apparent, although they were not completely discrete. The first cluster included exclusively IAR populations (Fènis, Pontboset, Sarre, Brusson and Champorcher), whereas the second cluster grouped ACW populations (Vens-1 and Vens-2) together with IAR populations Arnad and Morgex.

These two clusters were further resolved along the third coordinate (9.8% of the total variation). Within the first cluster, Fènis was clearly separated from two pairs of populations (Brusson–Champorcher and Pontboset–Sarre). Within the second cluster, Arnad was differentiated from Vens-1, which in turn was separated from the remaining populations. Overall, the PCoA highlights a structured genetic landscape characterized by both clear differentiation among populations and more subtle relationships within clusters.

### 3.3 Genetic relationships and differentiation among populations

**Figure 3:**
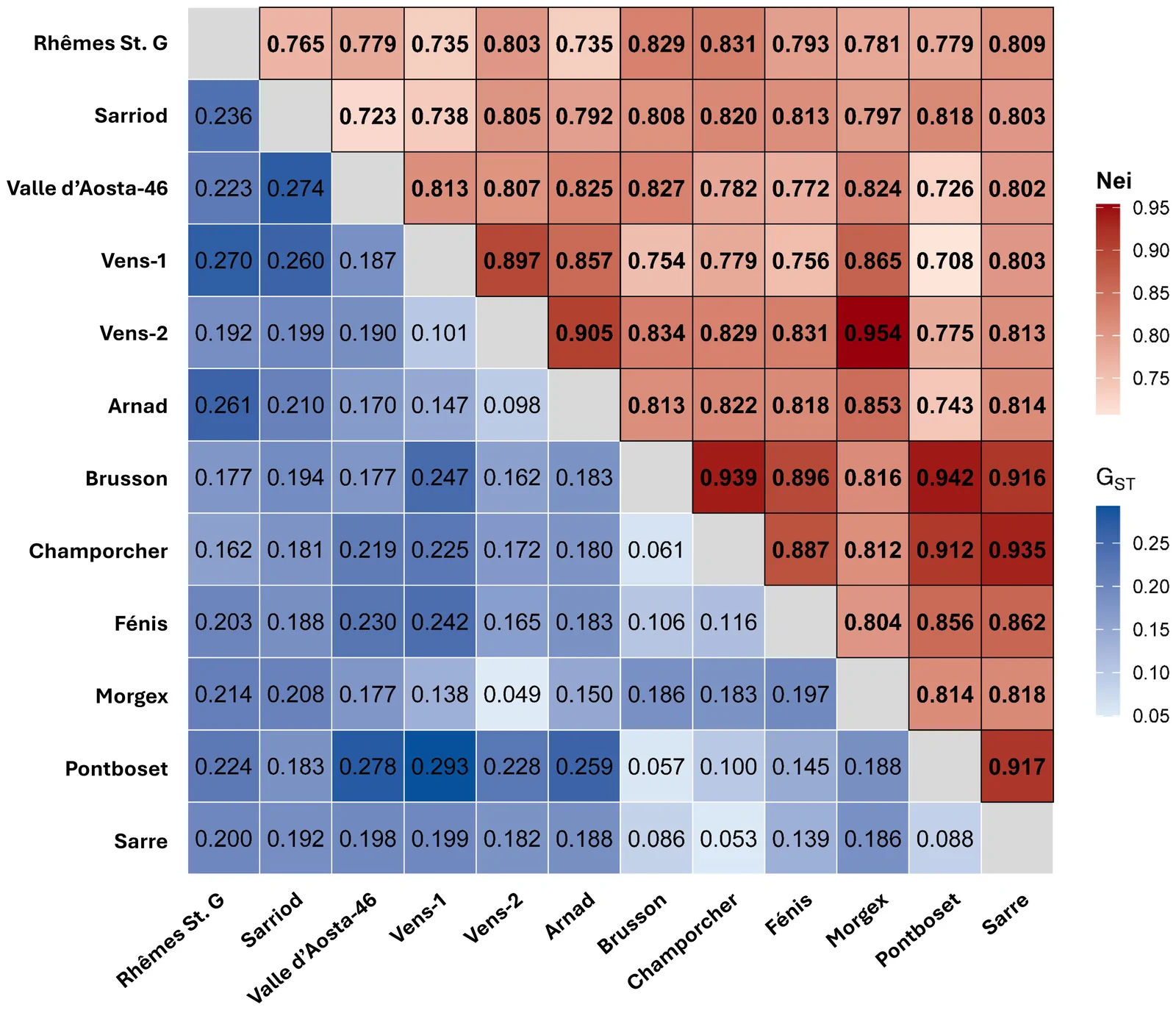
Heatmap of pairwise genetic relationships among rye populations. Values above the diagonal represent Nei’s genetic identity, while values below the diagonal represent pairwise G_ST_ estimates calculated from the filtered SNP dataset.

Pairwise genetic relationships based on Nei’s genetic identity and pairwise G_ST_ estimates were broadly consistent with the patterns observed in the multivariate analysis (**Figure 3**). Genetic identity values were generally high, often exceeding 0.75, indicating substantial similarity among several populations. However, notable variation was observed across population pairs, with some populations showing lower genetic similarity and greater differentiation. Pairwise G_ST_ values (**Figure 3**, below diagonal) showed variable levels of differentiation, with lower values observed among closely related population pairs. Groups of closely related populations were identified, such as Brusson–Pontboset and Champorcher–Sarre, which displayed high genetic identity and low G_ST_ values.

**Figure 4:**
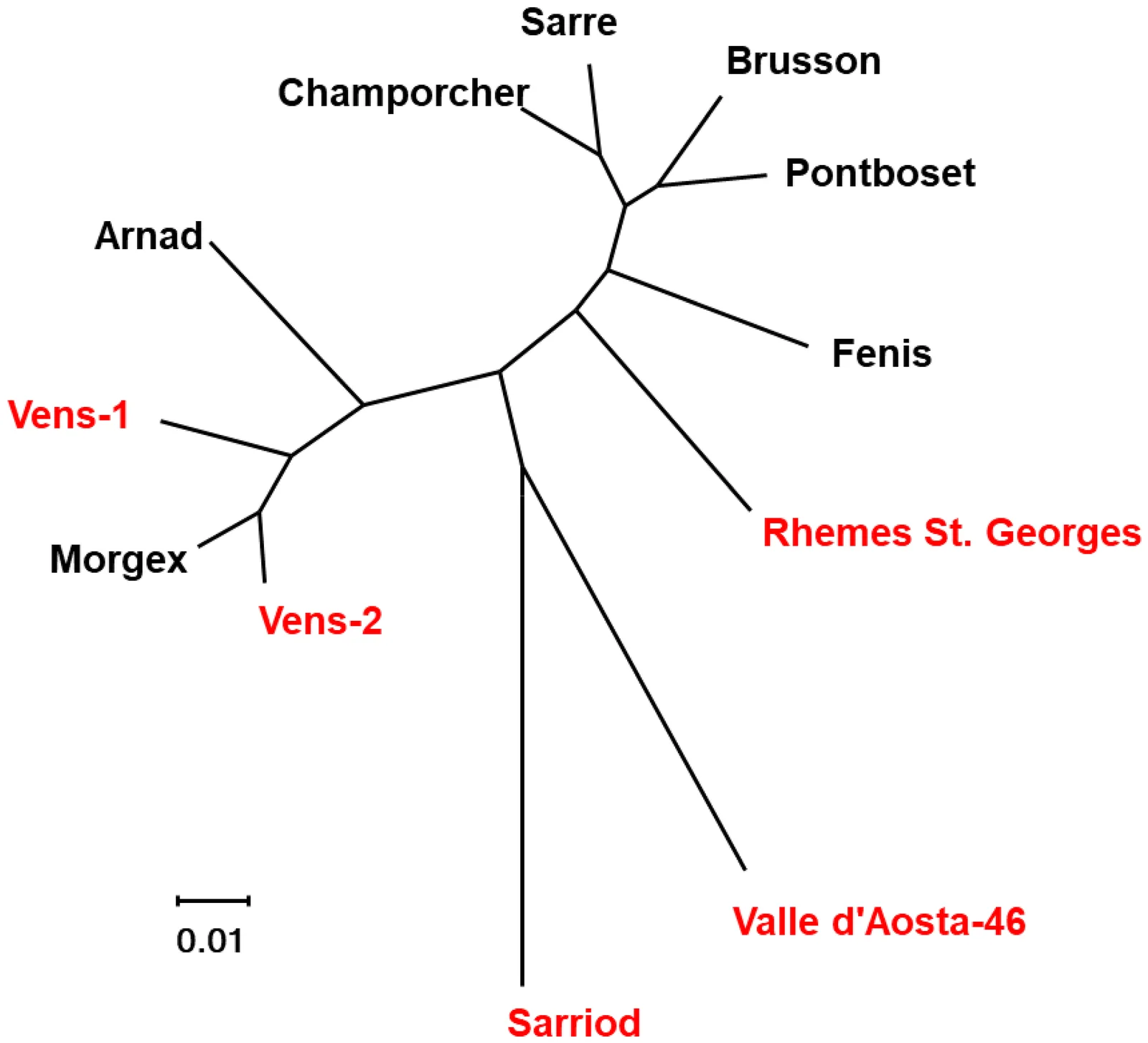
Neighbor-Joining dendrogram of rye populations based on Nei’s genetic distance, calculated as *D = −ln(I)*, where I is Nei’s genetic identity. Branch lengths are proportional to genetic distance. Populations collected in the 1980s (ACW) are highlighted in red, whereas populations collected in 2000 (IAR) are shown in black.

The Neighbor-Joining (NJ) dendrogram, based on Nei’s genetic distance, summarized the population relationships identified from the pairwise comparisons (**Figure 4**). Populations were grouped into distinct clusters, with some forming clearly separated branches, while others clustered together with short genetic distances. A subset of populations (Sarriod, Valle d’Aosta-46 and Rhêmes St. Georges) exhibited strong genetic differentiation, appearing as distinct lineages within the dendrogram. Conversely, several populations grouped into compact clusters, including Vens-1 and Vens-2 together with Morgex, as well as the pairs Brusson–Champorcher and Pontboset–Sarre.

Estimates of genetic differentiation based on Nei’s GST varied among the analyzed population groups (**Table 2**). Across all populations, GST was 0.254. When the two historical collections were analyzed separately, ACW populations showed a higher GST value (0.311) than IAR populations (0.180).

**Table 2:** Genetic diversity and differentiation parameters for subdivided rye populations.

Group	Sample size	H _T_	H _S_	G _ST_
ACW 1980 populations	5 pop. 80 plants	0.392	0.270	0.311
IAR 2000 populations	7 pop. 112 plants	0.306	0.251	0.180
**All populations**	**12 pop. 192 plants**	**0.354**	**0.264**	**0.254**

HT, total gene diversity (Nei, 1973); HS, gene diversity within populations; G_ST_, coefficient of genetic differentiation. Population groups correspond to ACW (1980s populations) and IAR (2000 populations).

### 3.4 Population structure

**Figure 5:**
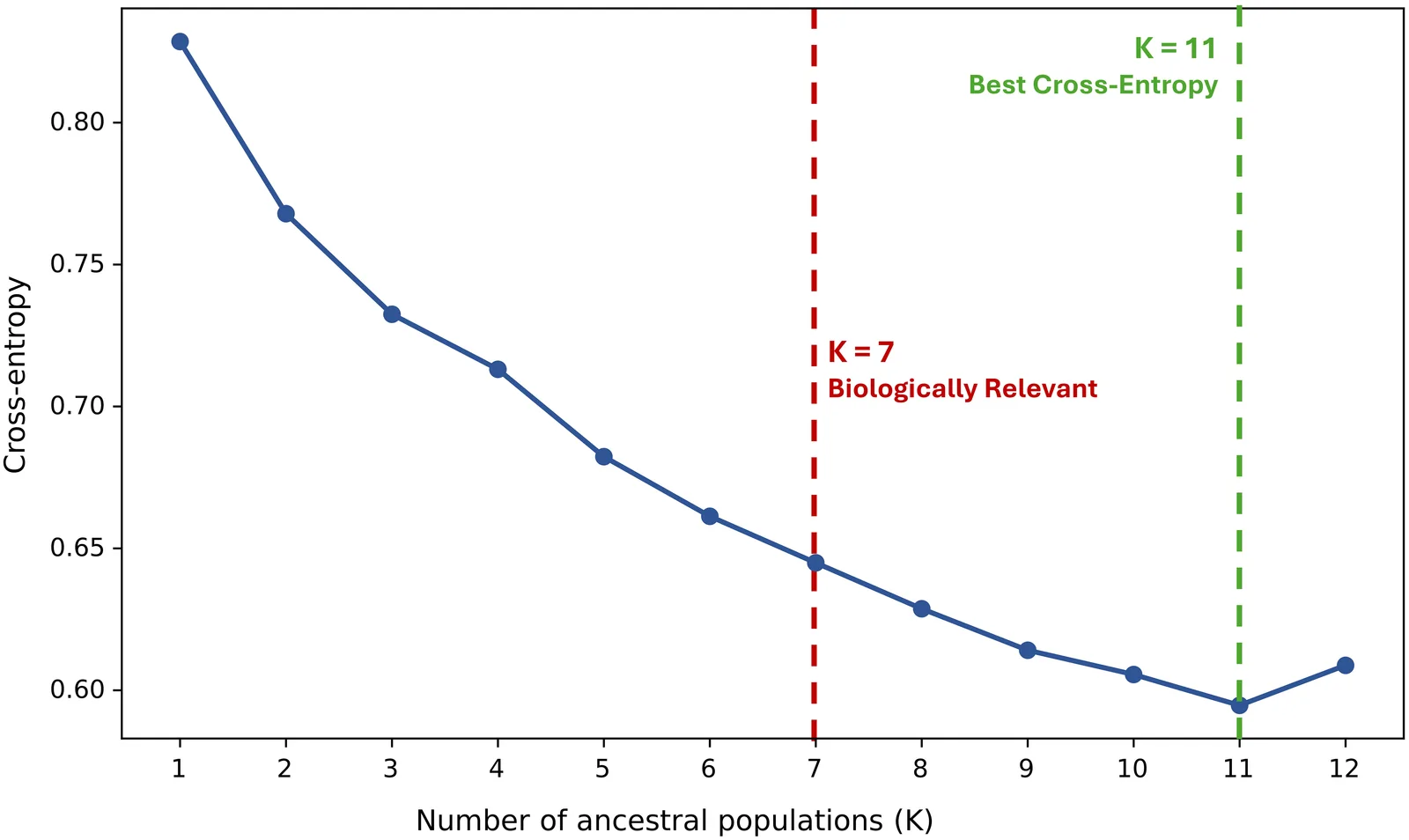
Cross-entropy criterion used to evaluate the number of ancestry components (K) in the LEA::snmf analysis. The best cross-entropy value across 15 replicate runs is shown for each K from 1 to 12; lower values indicate better predictive performance. The overall minimum was observed at K = 11. K = 7, indicated separately in the figure, was retained as a lower-complexity representation for visualization of the major ancestry patterns shown in **Figure 6**.

**Figure 6:**
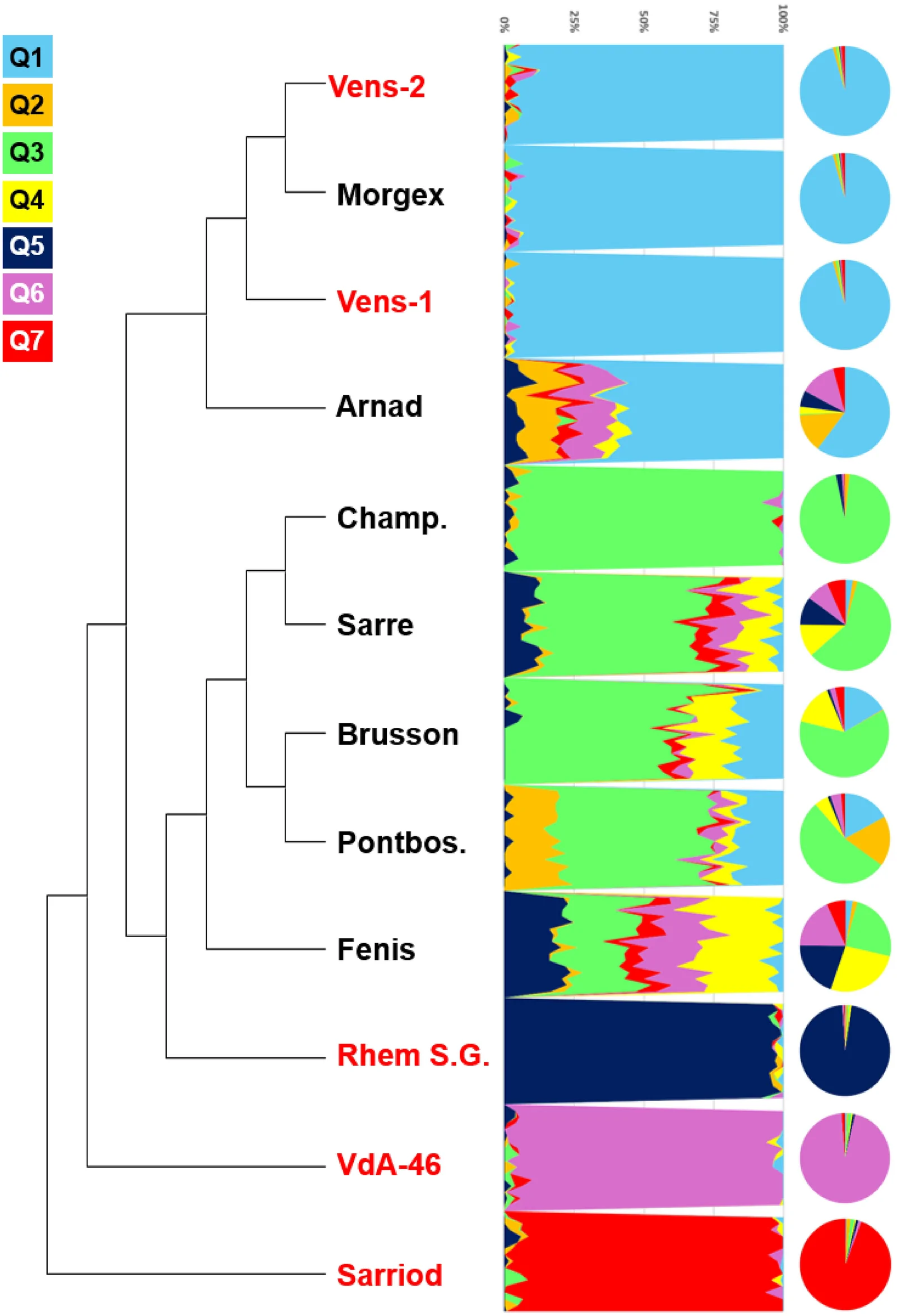
Integrated analysis of genetic relationships among rye populations based on UPGMA clustering based on pairwise G_ST_ values and individual ancestry coefficients inferred by LEA::snmf at K = 7. Although the minimum cross-entropy was observed at K = 11 (**Figure 5**), K = 7 is shown here as a lower-complexity representation of the major ancestry patterns. Numbers Q1–Q7 on the left correspond to the seven inferred ancestry components and match the colors used in the barplots. Populations collected in the 1980s (ACW) are highlighted in red, whereas populations collected in 2000 (IAR) are shown in black. Each horizontal bar represents one individual, and pie charts summarize mean ancestry proportions for each population.

Model-based clustering revealed hierarchical genetic structure across the tested range of K values (**Figure 5**). Cross-entropy generally decreased with increasing K and reached its minimum at K = 11, which therefore represented the best-supported solution according to this criterion. Higher K values, however, progressively subdivided ancestry components already apparent at lower K. For visualization of the major ancestry patterns and comparison with the population-level relationships identified by the other analyses, K = 7 was retained as a lower-complexity representation (**Figure 6**).

Individual assignment probabilities were heterogeneous. At K = 7, 113 of 192 individuals (58.85%) had Qmax ≥ 0.80, whereas 79 individuals (41.15%) had Qmax< 0.80 and were considered as showing mixed ancestry according to this operational threshold. Individuals with Qmax< 0.80 were concentrated in five populations: all individuals from Arnad, Sarre, Pontboset and Fénis (16/16 in each population), and 15 of 16 individuals from Brusson. These patterns are clearly visible in the individual ancestry barplots shown in **Figure 6**. The integrated analysis of population relationships and individual ancestry coefficients (**Figure 6**) showed broad correspondence between population-level UPGMA relationships and model-based ancestry patterns. However, the two approaches summarize different aspects of the data. The UPGMA tree represents average genetic relationships among populations, whereas the ancestry coefficients estimate the relative contribution of the seven inferred ancestry components for each individual. The ancestry barplots therefore revealed within-population heterogeneity and mixed ancestry patterns that are not directly quantified by the population-level UPGMA tree.

## 4 Discussion

### 4.1 Genetic diversity and population structure

The genomic characterization performed in this study provides a high-resolution view of the genetic diversity and structure of rye populations from the Aosta Valley. The results revealed substantial genetic variation within populations, as expected for an outcrossing species, and heterogeneous differentiation among populations (Persson and von Bothmer, 2002; Hagenblad et al., 2016; Adamo et al., 2021). The overall *G_ST_* estimate indicated that genetic variation was predominantly maintained within populations, although a relevant component of differentiation was also detected among populations. These estimates refer specifically to the filtered SNP dataset used in this study and should therefore be interpreted primarily in comparative terms.

The combined evidence from multivariate, distance-based and model-based analyses revealed a structured genetic landscape. Sarriod, Valle d’Aosta-46 and Rhêmes St. Georges occupied relatively distinct positions across the different analyses. In contrast, other populations showed closer genetic relationships, including Vens-1 and Vens-2 with Morgex, as well as the pairs Brusson–Champorcher and Pontboset–Sarre. These results highlight the coexistence of differentiated populations, closely related groups and within-population heterogeneity within the analyzed germplasm.

### 4.2 Temporal patterns of genetic diversity

A key result of this study is the clear difference in genetic diversity between populations collected in the 1980s (ACW) and those collected in 2000 (IAR). All diversity parameters, including H_T_, PL, n_a_, n_e_ and Shannon’s index, were consistently higher in ACW populations compared to IAR populations. In addition, ACW populations showed a higher level of genetic differentiation (G_ST_ = 0.311) than IAR populations (G_ST_ = 0.180), indicating stronger genetic structuring in the older collection. However, because the two collections do not necessarily represent repeated sampling of the same localities, these differences cannot be attributed exclusively to temporal changes.

These differences may reflect a reduction of genetic variability in the later collection, potentially associated with the sharp decline of rye cultivation, reduced population sizes and/or increased homogenization of seed material. However, differences in sampling localities and seed-management histories may also have contributed to the observed pattern. Agricultural Census data for Valle d’Aosta are consistent with the first interpretation, showing a 94% reduction in recorded rye cultivation area between 1982 and 2000, from 29.2 to 1.8 ha. This suggests that rye may have progressively disappeared from professional agricultural production, persisting mainly among a limited number of hobby or small-scale growers and thus partly escaping statistical detection. The consistency of this pattern across all diversity indices supports the robustness of this interpretation. Previous studies based on AFLP markers reported comparable patterns of high within-population variability and moderate differentiation (Chwedorzewska et al., 2002; Adamo et al., 2021).

On a broader scale, recent genomic studies on rye populations in the Western Alps have highlighted a general trend toward genetic homogenization. In this context, the present study provides a complementary perspective by focusing on a well-defined set of populations from the Aosta Valley. While the overall patterns are consistent with regional trends, the results also demonstrate the persistence of distinct genetic entities within local germplasm.

### 4.3 Implications for conservation and value of K-seq

The identification of both genetically distinct and closely related populations has important implications for the conservation of rye genetic resources. Populations showing clear genetic differentiation may represent reservoirs of distinctive allelic variation and should be considered in conservation programs (Blair, 1996; Hoban et al., 2020; von Takach et al., 2024). Conversely, the presence of highly similar populations suggests potential redundancy within the germplasm, indicating that conservation strategies could be optimized by focusing on representative populations while maintaining sufficient variability (Schoen and Brown, 1993; Odong et al., 2013).

The high level of intra-population variability further emphasizes the importance of maintaining adequate population sizes and implementing appropriate sampling strategies during seed regeneration, in order to avoid genetic drift and loss of diversity (Chebotar et al., 2003; Graudal et al., 2014; Martina et al., 2025; Morales‐González et al., 2025; Mergeay et al., 2026). This is particularly relevant for open-pollinated species such as rye, where genetic variation within populations plays a key role in adaptive potential (Hagenblad et al., 2016). The substantial within-population variation observed here is also consistent with the hidden genic diversity reported in heterogeneous rye accessions by Hawliczek et al. (2020). Their pooled deep-sampling approach complements the present individual-level reduced-representation analysis by emphasizing the importance of adequate sampling for capturing diversity within rye populations.

In this context, K-seq generated an informative reduced-representation SNP dataset that allowed broad and fine-scale genetic structure to be investigated in this rye panel. Its practical features include the use of species-specific, k-mer-derived primers, a relatively simple library workflow, and independence from restriction-enzyme digestion, which may be advantageous when working with large and repeat-rich genomes (Ziarsolo et al., 2021; Martina et al., 2023). However, marker representation depends on primer selection, and the resulting SNP panel does not constitute an unbiased sample of the whole genome. Therefore, the present study illustrates the utility of K-seq in this dataset but does not represent an independent validation of the method or a comparison with GBS or ddRAD-seq.

## 5 Concluding remarks

This study provides an updated genomic characterization of rye populations from the Aosta Valley, highlighting the coexistence of differentiated populations, groups of closely related populations, and substantial within-population variation. The comparison between the two historical collections also revealed differences in genetic diversity and differentiation, although these patterns cannot be attributed exclusively to temporal changes because the collections did not necessarily originate from the same localities.

The results underline the importance of conserving genetically diverse and differentiated populations while maintaining adequate within-population variation. More broadly, the study illustrates the utility of combining historical germplasm collections with reduced-representation SNP genotyping to support the characterization, conservation, and sustainable management of plant genetic resources in marginal environments.

## Data Availability

The original contributions presented in the study are publicly available. The raw sequencing data generated in this study are deposited in the NCBI Sequence Read Archive under BioProject accession PRJNA1507426. The filtered SNP matrix is provided in **Supplementary Table S1**. Sample metadata, sample-level sequencing statistics, LEA cross-entropy values, K = 7 ancestry coefficients, marker distribution and filtering criteria are provided in **Supplementary Table S2**.

## Supplementary material

Supplementary Figure 1 Cattell scree plot of the Principal Coordinates Analysis (PCoA) based on the filtered SNP dataset. Eigenvalues are plotted against successive principal coordinates to illustrate the distribution of genetic variation across dimensions. The elbow observed around coordinate 18 provides a descriptive indication of the dimensionality of the genetic variation represented in the dataset.

Supplementary Table 1 Supporting datasets and analytical information for the Aosta Valley rye populations. The Excel workbook contains the supplementary data generated and used in this study, including individual sample identifiers and population assignments, collection group (ACW or IAR), collection year and locality information; sample-level sequencing statistics; the filtered SNP dataset used for population genetic analyses; chromosome-level SNP counts, marker densities and representation in chromosome-terminal regions; cross-entropy values obtained from the 15 replicate LEA::snmf runs for each K value from 1 to 12.
